# Tenofovir is associated with increased tubular proteinuria and asymptomatic renal tubular dysfunction in Ghana

**DOI:** 10.1186/s12882-015-0192-4

**Published:** 2015-12-01

**Authors:** David R. Chadwick, Fred S. Sarfo, Elaine S. M. Kirk, Dorcas Owusu, George Bedu-Addo, Victoria Parris, Ann Lorraine Owusu, Richard Phillips

**Affiliations:** Centre for Clinical Infection, James Cook University Hospital, Middlesbrough, TS4 3BW UK; Kwame Nkrumah University of Science & Technology, Kumasi, Ghana; Komfo Anokye Teaching Hospital, Kumasi, Ghana; University of Leicester, Leicester, UK

**Keywords:** HIV, Kidney, Renal, TDF, Tubular, Africa, Antiretroviral therapy

## Abstract

**Background:**

HIV infection is associated with increased risk of renal dysfunction, including tubular dysfunction (TD) related to antiretroviral therapy (ART). Tenofovir disoproxil fumarate (TDF) is becoming available for ART in sub-Saharan Africa, although data on its long-term safety there is limited. We aimed to study the prevalence of HIV-associated renal dysfunction in Ghana and explore associations between proteinuria or TD and potential risk factors, including TDF use.

**Methods:**

A single-centre cross-sectional observational study of patients taking ART was undertaken. Creatinine clearance (CrCl) was calculated and proteinuria detected with dipsticks. Spot urinary albumin and protein:creatinine ratios (uACR/uPCR) were measured and further evidence of TD (defined as having two or more characteristic features) sought. Logistic regression analysis identified factors associated with proteinuria or TD.

**Results:**

In 330 patients, of whom 101 were taking TDF (median 20 months), the prevalence of CrCl < 60ml/min/1.73m^2^, dipstick proteinuria and TD was 7 %, 37 % and 15 %. Factors associated with proteinuria were baseline CD4-count [aOR 0.86/100 cell increment (95 % CI, 0.74–0.99)] and TDF use [aOR 2.74 (95 % CI, 1.38–5.43)]. The only factor associated with TD was TDF use [aOR 3.43 (95 % CI, 1.10–10.69)]. In a subset with uPCR measurements, uPCRs were significantly higher in patients taking TDF than those on other drugs (10.8 *vs.* 5.7 mg/mmol, *p* < 0.001), and urinary albuin:protein ratios significantly lower (0.24 *vs.* 0.58, *p* < 0.001).

**Conclusions:**

Both proteinuria and TD are common and associated with TDF use in Ghana. Further longitudinal studies to determine whether proteinuria, TD or TDF use are linked to progressive decline in renal function or other adverse outcomes are needed in Africa.

## Background

HIV-infected individuals are at increased risk of renal dysfunction due to a number of causes including HIV-associated nephropathy (HIVAN), HIV immune complex glomerulonephritis and antiretroviral therapy (ART) [1–4]. The use of ART has decreased HIV-associated renal dysfunction and progression to end stage renal disease (ESRD) [5–8], but renal dysfunction remains common amongst HIV-infected patients. There is a higher prevalence of renal dysfunction and ESRD among the African-American population (largely of West African descent) compared to Caucasians [9–11]. Genetic polymorphisms [12], higher prevalence of diabetes mellitus and hypertension, and other co-morbidities including hepatitis B virus (HBV), schistosomiasis and malaria infections ensure that renal dysfunction is common in West Africans regardless of HIV status. HIVAN is the most common HIV-related renal pathology in sub Saharan Africa [13, 14]. It appears that the prevalence of HIV-related renal dysfunction, defined by estimated glomerular filtration rate (eGFR) <60 ml/min, may be higher among ART-naïve West Africans compared with those from East and Southern Africa [7, 13, 15–19].

The nucleotide reverse transcriptase inhibitor tenofovir disoproxil fumarate (TDF), which is active against HBV in addition to HIV, is now widely used in Africa. TDF is excreted through a combination of glomerular filtration and active tubular secretion, with uptake in the proximal tubule and secretion into the tubular space [20]. Initial clinical trials described a favourable renal safety profile [21, 22], however there were rare post-marketing reports of Fanconi’s syndrome (proximal tubulopathy) leading to glucosuria, phosphaturia, aminoaciduria and bicarbonate wasting [23, 24]. Subsequent studies have found that TDF use causes a small but significant decrease in creatinine clearance (a surrogate for glomerular filtration) in both HIV positive and negative populations [25, 26]. There has been increasing interest in TDF-associated tubular dysfunction (TD), the duration of TDF exposure appearing to play a part, with cumulative TDF exposure associated with increased risk of proximal TD in adults [27–30]. Genetic polymorphisms may cause a predisposition for TDF associated TD [20]. Little is known about the prevalence of TD in Africa as it is difficult to screen for this complication in resource-poor areas, and few African studies have investigated markers of TD. We therefore undertook this study to determine the prevalence of renal dysfunction including TD in HIV-infected individuals taking ART in Ghana and examine potential associated risk factors including TDF exposure.

## Methods

This was an observational cross-sectional single-centre study involving HIV-infected patients taking ART. This study was conducted at the Komfo Anokye Teaching Hospital (KATH) in Kumasi, Ghana. The Committee on Human Research Publications and Ethics at KATH approved the study. Patients were initiated on either zidovudine or stavudine with lamivudine, plus either nevirapine or efavirenz prior to 2010 when TDF became available. After this a small proportion of patients, included those identified with hepatitis B co-infection, were initiated on (or switched to) TDF. All patients ≥18 years old attending the HIV clinic who had been taking ART for at least 6 months and who gave informed consent during the study period were screened for renal dysfunction by urine dipsticks and serum creatinine. Demographic, medical and laboratory data was collected from case notes. Individuals with known causes of renal impairment or urinary tract infections (positive dipstick for leukocytes and nitrites), were excluded. Patients with ‘one plus’ or more of protein or glucose on dipsticks were considered positive for proteinuria or glycosuria, the latter only if a blood glucose was <9 mmol/L. To further define the level and characteristics of proteinuria in a (randomly-selected) subset of individuals with proteinuria, spot urine protein:creatinine (uPCR), urine albumin:creatinine (uACR) and urinary albumin:protein (uAPR) ratios were calculated. Creatinine clearance (CrCl) was calculated using the Cockroft-Gault equation, based on local practice patterns.

In a subset of patients – all those who had sufficient volumes of urine and blood specimens - fractional phosphate and urate excretion was measured, in addition to glycosuria and uAPR, to identify TD. TD was defined as having two or more of the following: fractional phosphate excretion >18 %, fractional urate excretion >15 %, normo-glycaemic glycosuria, proteinuria (uPCR > 20 mg/mmol) with uAPR < 0.4. Descriptive results of continuous variables were expressed as medians and interquartile ranges. Continuous variables were compared using Student’s *t* test or Wilcoxon rank sum test, as required. For the comparison of proportions, the Chi-squared test was used, with Fisher’s corrections applied when needed. Univariate and multivariate logistic regression analyses were performed to identify factors associated with proteinuria or TD. Parameters with *p* < 0.1 in the univariate analysis were entered into a stepwise multivariate analysis. All statistics were conducted using SAS, version 9.3.

## Results

In total, 367 patients were screened for evidence of renal dysfunction with urine dipsticks and blood creatinine measurement, and 37 excluded due to evidence of urinary tract infection or other causes of renal impairment, including diabetes and hypertension. Of the 330 remaining, who had been taking ART for a median of 24 (IQR 15–48) months, 101 were taking TDF for a median of 20 (12–14) months, with the remainder taking either stavudine or zidovudine with lamivudine. The characteristics of this population are shown in Table 1. Demographic characteristics were similar amongst patient taking or not taking TDF. Proteinuria was common, found in 37 % of the entire cohort, and confirmed in 20/167 (12 %) patients with uPCRs >20 mg/mmol. 7 % of patients had CrCl <60 ml/min/1.73m^2^. TD was found in 12 of 82 (15 %) patients who were evaluated. Patients on TDF were more likely to be HBV co-infected (HBsAg+), have glycosuria and proteinuria on dipsticks, had significantly higher uPCRs (10.8 vs 5.7 mg/mmol, *p* < 0.001) and lower uAPRs (0.24 vs 0.58, *p* < 0.001), with 35 % of those on TDF (*vs.* 6 % not on TDF) satisfying the criteria for TD.

**Table 1:** Characteristics of the study population

	Taking TDF	Not taking TDF	All patients	*p*
	(*n* = 101)	(*n* = 229)	(*n* = 330)	
Female, No. (%)	82 (81)	165 (72)	247 (75)	0.08
Age, Median (IQR), years	38 (34–46)	40 (36–46)	39 (35–46)	0.09
BMI, Median (IQR), kg/m^2^	22.8 (21.5–25.3)	22.9 (20.3–27.1)	22.9 (20.5-26.6)	0.11
Nadir CD4 Median (IQR), cells/μl	164 (94–265)	186 (76–297)	185 (79–297)	0.79
Current CD4 Median (IQR), cells/μl	545 (337–679)	456 (335–630)	463 (329–640)	0.22
CrCl^d^, Median (IQR), ml/min/1.73m^2^	99 (78–118)	96 (77–126)	95 (77–118)	0.21
CrCl < 60 ml/min/1.73 m^2^, No (%)	5 (5)	19 (8)	24 (7)	0.28
Efavirenz-based ART, No. (%)^b^	71 (70)	147 (64)	218 (66)	0.28
Duration on tenofovir, Median (IQR), months	20 (12–24)			
HBsAg positive^a^, No. (%)	20/56 (36)	8/101 (8)	28/157 (18)	0.0004
Proteinuria (dipstick), No. (%)	62 (61)	73 (32)	123 (37)	<0.0001
uPCR^b^, Median (IQR), mg/mmol	10.8 (6.8–15.1)	5.7 (3.3–10.4)	7.7 (5.2–12.9)	<0.0001
uACR^b^, Median (IQR), mg/mmol	2 (0.58–2.4)	2.1 (1.0–4.6)	2 (0.7–3.4)	0
uAPR^e^, Median (IQR), mg	0.24 (0.1–0.4)	0.58 (0.4–0.6)	0.43 (0.2–0.6)	0.0006
Fractional phosphate excretion > 18 %^d^, No. (%)	2/28 (7)	6/54 (11)	8/82 (10)	0.57
Fractional urate excretion > 15 %^d^, No. (%)	1/28 (4)	4/54 (7)	5/82 (6)	0.49
Glycosuria (dipstick), No. (%)	24 (24)	5 (2)	29 (9)	<0.0001

*TDF* tenofovir disoproxil fumarate, *BMI* body mass index, *CrCl* estimated creatinine clearance (by Cockcroft Gault formula), *uPCR* urinary protein:creatinine ratio, *uACR* urinary albumin:creatinine ratio, *uAPR* urinary albumin:protein ratio. ^a^ *HBsAg* hepatitis B surface antigen (based on a subset of 157 patients)

^b^Based on subset of 161 patients. ^c^Most other patient were taking nevirapine. ^d^ Based on subset of 82 patients

^e^Where uPCR > 20 mg/mmol

Univariate and multivariate analyses of factors associated with dipstick proteinuria (in the entire population) and TD (in a subset of 82 patients) are shown in Table 2. Younger age and lower baseline CD4 count were marginally associated with proteinuria, odds ratio (OR) 0.77 (95 % CI, 0.59–1.00) and 0.88 (0.77–1.01) respectively, as was TDF use, OR 1.82 (1.12–2.95). Factors independently associated with proteinuria were lower baseline CD4 count, adjusted odds ratio (aOR) 0.86 per 100 cell increment (0.74–0.99), and TDF use, aOR 2.74 (1.38–5.43). In a sensitivity analysis, use of TDF was significantly associated with proteinuria, unadjusted OR of 3.56 (1.21-10.50), *p* = 0.02, where only patients with confirmed uPCR > 20 (not dipstick-only proteinuria) were included. Similar results were also found where only patients with dipstick proteinuria >1+ were included. Although lower age was associated with TD in univariate analysis, the only factor associated with TD in both univariate and multivariate analyses was TDF use, aOR 3.43 (1.10–10.69). Of note, duration of TDF therapy was not significantly associated with odds of proteinuria, uPCR, or TD.

**Table 2:** Predictors of proteinuria and tubular dysfunction in HIV-infected Ghanaians

Variable	Proteinuria				Tubular dysfunction			
	OR (95 % CI)	*p*	aOR (95 % CI)	*p*	OR (95 % CI)	*p*	aOR (95 % CI)	*p*
Age/10 years	0.77 (0.59–1.00)	0.05	0.74 (0.54–1.01)	0.06	0.58 (0.35–0.98)	0.04	0.62 (0.36–1.06)	0.08
Baseline CD4/100 cells/mm^3^	0.88 (0.77–1.01)	0.07	0.86 (0.74–0.99)	0.04	1.11 (0.88–1.40)	0.38		
Gender								
Female	1.05 (0.63–1.78)	0.84			0.96 (0.35–2.61)	0.93		
Male	1				1			
BMI /5 kg/m^2^	0.88 (0.69–1.12)	0.29			0.83 (0.50–1.38)	0.47		
Each year on ART	0.95 (0.84–1.07)	0.38			0.92 (0.69–1.21)	0.53		
Use of TDF								
Yes	1.82 (1.12–2.95)	0.01	2.74 (1.38–5.43)	0.004	3.83 (1.24–11.77)	0.02	3.43 (1.10–10.69)	0.03
No	1		1		1		1	
Current CD4/100 cells/mm^3^	0.98 (0.89–1.08)	0.65			1.09 (0.93–1.29)	0.29		
Duration on TDF/1yr	1.00 (0.97–1.03)	0.86			1.13 (0.73–1.76)	0.57		
HBV Serology								
HBsAg +	0.81 (0.36–1.85)	0.62			1.93 (0.49–7.56)	0.34		
HBsAg -	1				1			

*OR* odds ratio, *aOR* adjusted odds ratio, *TDF* tenofovir disoproxil fumarate, *BMI* body mass index, *ART* antiretroviral therapy, *HBV* hepatitis B virus

## Discussion

Previous studies of populations taking TDF, including some in Africa, have shown that significant declines in renal function are rare [7, 31, 32]. However only the DART study, based in East and Southern Africa, has followed patients beyond 2 years [29], and to our knowledge no studies have assessed TD in addition to declines in estimated glomerular filtration rates (eGFR)/CrCl or proteinuria in Africa. We postulated that proteinuria may be more frequent in West Africans taking ART due to a genetic predisposition and other factors such as HBV co-infection, which is common in Ghana [33] and has been associated with renal dysfunction in HIV-infected patients [34]. We also postulated that the phasing out of stavudine and zidovudine in the Ghana ART programme, and their gradual replacement with TDF from 2010, might lead to an increase in TD in patients on TDF-based ART. Our study has shown strong evidence of both TD and proteinuria being associated with TDF use. It has also demonstrated that whilst CrCl < 60mls/min is relatively rare in this population, TD is common (found in 15 % of all patients and 35 % of those on TDF) as is proteinuria, which was found in over one third of the population studied. Cross-sectional studies in European populations have identified between 7–22 % of patients on ART having evidence of TD [20, 28–30], using different definitions of TD, which is similar to the overall rate found in Ghana however lower than that found in patients taking TDF. Given that use of protease inhibitors has also been associated with TD, since no patients receiving protease inhibitors had features of TD, it is likely most ART-related TD was due to TDF alone [29, 35]. As in other studies, very few of the patients meeting our definition of TD had evidence of clinically-significant TD or Fanconi syndrome, and the long-term significance of this finding is uncertain.

Hardly any studies outside Africa have identified such a high proportion of ART-treated patients having proteinuria as ours. High rates of proteinuria have been noted in two other studies in Africa, however neither study distinguished the type of proteinuria in terms of albumin and total protein [12, 36]. This study demonstrated that in patients with significant proteinuria, those taking TDF had a lower albumin:protein ratios which is characteristic of tubular rather than glomerular dysfunction [37], indicating that HIVAN was probably not the predominant pathology causing renal dysfunction. Interestingly, lower age was associated with proteinuria in our study, in contrast to older age being more often associated with proteinuria, although this might be explained by orthostatic proteinuria which is common in young adults. Given increasing evidence that an alternative form of tenofovir, tenofovir alafenamide (TAF), is less nephrotoxic than TDF [38], and is likely to replace TDF over the next decade, it is perhaps less likely that significant numbers of patients will develop TDF-associated renal dysfunction or chronic kidney disease (CKD).

Limitations of this study include the relatively small sample of patients who were tested for features of TD and numbers who had available HBV serology, limiting the power of the analyses to demonstrate correlations of postulated risk factors with TD. Given the strong association between TDF use and HBsAg + status, this may also have introduced confounding by indication. Furthermore, the prevalence of proteinuria and TD may have been over-estimated given that this was a cross-sectional study without repeated measurement, and in just over half of patients proteinuria was not confirmed or quantified by measuring uPCR.

## Conclusions

In summary this study has shown that both TD and proteinuria are common amongst Ghanaians taking ART, and TDF use is independently associated with both of these outcomes. Further studies are needed assessing clinical outcomes including decline in eGRF/CrCl and mortality in this population to evaluate the clinical significance of these findings.

## Abbreviations

HIV: Human immunodeficiency virus
HIVAN: HIV-associated nephropathy
ART: Antiretroviral therapy
ESRD: End stage renal disease
HBV: Hepatitis B virus
TD: Tubular dysfunction
